# Beyond the canon: emerging modalities of regulated cell death

**DOI:** 10.1038/s41418-026-01686-w

**Published:** 2026-02-11

**Authors:** Daolin Tang, Gerry Melino, Guido Kroemer

**Affiliations:** 1 Department of Surgery, UT Southwestern Medical Center, Dallas, TX USA; 2 Department of Experimental Medicine, TOR, University of Rome Tor Vergata, Rome, Italy; 3 Centre de Recherche des Cordeliers, Université Paris Cité, Sorbonne Université, Inserm, Paris, France; 4 Centre de Recherche des Cordeliers, Équipe Labellisée par la Ligue contre le cancer, Paris, France; 5 Institut Gustave Roussy, INSERM US23/CNRS UAR 3655, Metabolomics and Cell Biology Platforms, UMS AMMICa, Université Paris-Saclay, Villejuif, France; 6 Department of Biology, Institut du Cancer Paris CARPEM, Hôpital Européen Georges Pompidou AP-HP, Paris, France

**Keywords:** Proteolysis, Cell death and immune response

The field of regulated cell death (RCD) has expanded in punctuated bursts—from apoptosis and necroptosis to pyroptosis, ferroptosis, and more recently, triaptosis [1–3]. In 2025, four landmark studies further reshaped this landscape by identifying mechanistically distinct death programs: necrosis by sodium overload (NECSO), spectosis, floatptosis, and mitoxyperilysis (Table 1). Each pathway features a unique initiating stimulus, morphological signature, and molecular execution mechanism, collectively illustrating how diverse physiological and pathological stresses can drive equally diverse cellular demise. This further expands the modalities of death originally described in the Nomenclature Committee on Cell Death [4]. Here, we highlight the defining characteristics of these newly described modalities and discuss what they reveal about the evolving logic of RCD.

**Table 1:** Comparative summary of four newly reported cell death modalities (2025).

Death Modality	Primary Inducer/Trigger	Representative Models	Morphological Features	Key Molecular Mechanisms/Determinants	Inhibition/Rescue Strategies
NECSO (Necrosis by sodium overload)	NC1 activation of human TRPM4 → Na^+^ overload	Human cancer cell lines (MCF7, MB468, HCC1143, PC3); TRPM4-expressing HeLa	Cell ballooning, organelle swelling, osmotic lysis	TRPM4-dependent Na^+^ influx, Cl^−^ entry, osmotic edema	*TRPM4* KO; 9‑phenanthrol; Na^+^-free medium; mannitol
Spectosis (Programmed RBC death via caspase‑8)	Complement MAC activation → miniNLRP3–caspase‑8 signaling	Human mature RBCs; complement-driven hemolysis	Discocyte → echinocyte → spherocyte → ghost	miniNLRP3–ASC–caspase‑8 axis; β‑spectrin proteolysis	Z‑VAD‑FMK; Z‑IETD‑FMK; MCC950 (partial)
Floatptosis (BCV‑induced vacuolization death)	*Bergeyella cardium* variant infection; OMVs; β‑barrel proteins	BMDMs, iBMDMs, L929, AC16 cells	Large fused LAMP1^+^/Rab7^+^ vacuoles; cytoplasmic compression	SLC9A9‑dependent vacuole fusion; lysosomal–endosomal remodeling	Amiloride; *SLC9A9* deficiency
Mitoxyperilysis (Mito–PM contact oxidative lysis)	Innate immune activation + carbon starvation	Primary BMDMs; tumor models	Sustained mitochondria–PM contacts; localized peroxidation; rupture	GSH depletion; BAX/BAK/BID; mTORC2‑regulated contact duration	mTOR inhibition; GSH supplementation; carbon sources

**Fig. 1:**
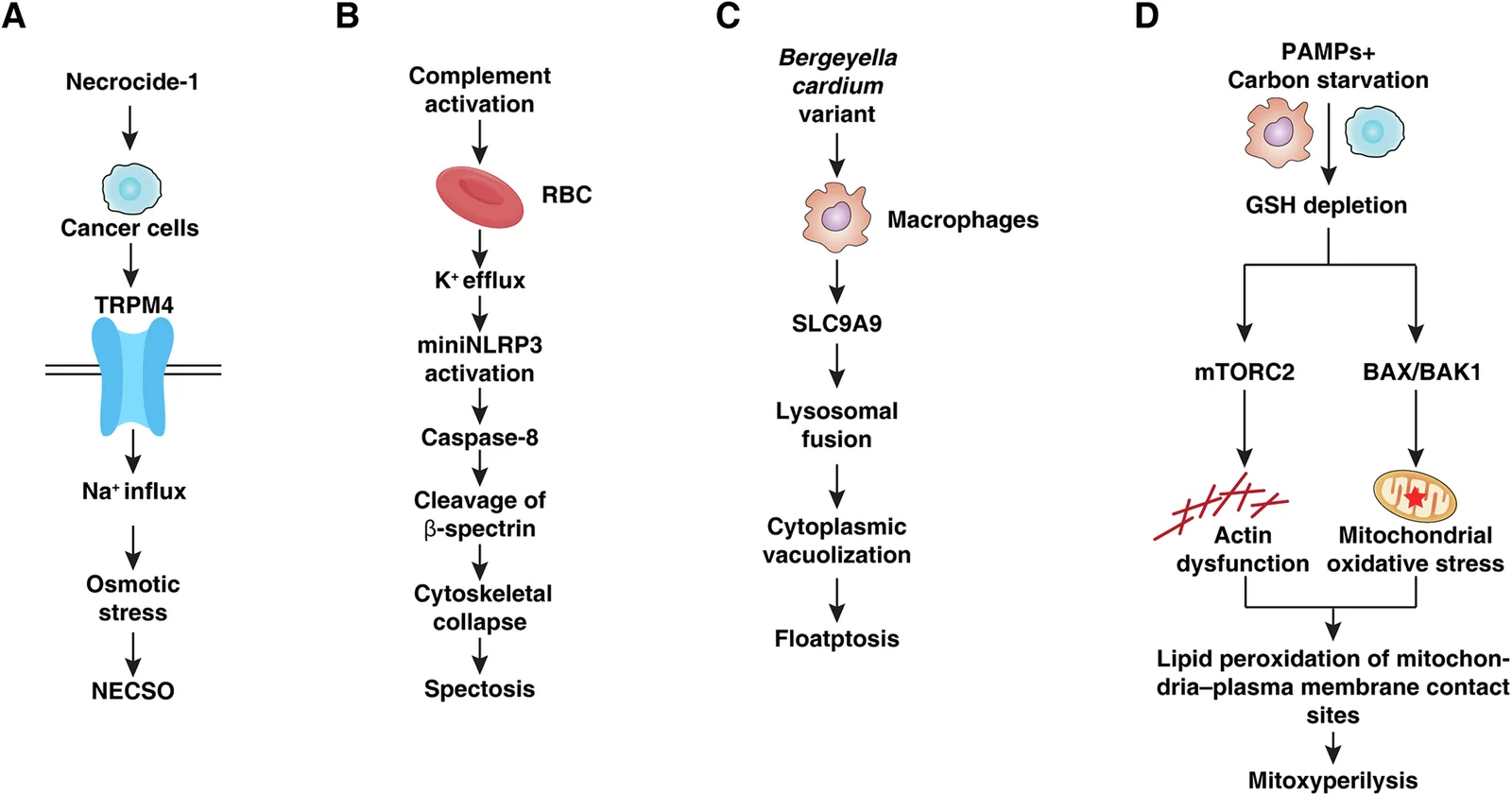
Mechanistic overview of recently described regulated cell-death modalities. **A** NECSO: In cancer cells, necrocide-1 induces TRPM4 activation, leading to Na⁺ influx, osmotic stress, and subsequent necrotic cell swelling culminating in NECSO. **B** Spectosis: Complement activation on red blood cells triggers K⁺ efflux and mini-NLRP3 activation, resulting in caspase-8–mediated cleavage of β-spectrin, cytoskeletal collapse, and spectrin-dependent cell death termed spectosis. **C** Floatptosis: Infection with *Bergeyella cardium* variant in macrophages induces SLC9A9-dependent lysosomal fusion, cytoplasmic vacuolization, and a distinct vacuole-associated death program defined as floatptosis. **D** Mitoxyperilysis: Combined pathogen-associated molecular patterns (PAMPs) and carbon starvation cause glutathione (GSH) depletion, activating parallel mTORC2-driven actin dysfunction and BAX/BAK1-mediated mitochondrial oxidative stress. These signals converge to promote lipid peroxidation at mitochondria–plasma membrane contact sites, leading to mitoxyperilysis.

The first of the newly recognized modalities, NECSO, centers on dysregulated sodium homeostasis rather than on more familiar mediators such as Ca²⁺, Fe²⁺, or reactive oxygen species (ROS) [5] (Fig. 1A). Fu et al. identified the small molecule necrocide-1 (NC1), which binds to and persistently activates human transient receptor potential cation channel subfamily M member 4 (TRPM4), a Ca²⁺-activated monovalent cation channel. Sustained TRPM4 opening drives overwhelming Na⁺ influx, accompanied by compensatory chloride entry and osmotic swelling that culminates in catastrophic membrane rupture. TRPM4 deletion abolishes Na⁺ influx and cell death, and the striking species specificity—NC1 strongly activates human but not mouse TRPM4—highlights the structural precision required for this process. Morphologically, NECSO is defined by rapid cell ballooning, organelle swelling, and membrane depolarization, hallmarks of an ionic catastrophe rather than a classical programmed pathway. Importantly, inhibitors of apoptosis, necroptosis, pyroptosis, ferroptosis, or autophagy do not prevent NECSO, underscoring its mechanistic independence. The discovery of NECSO reframes sodium overload, traditionally considered a secondary event in edema or ischemia, as a primary and self-sufficient execution mechanism.

A second conceptual shift emerged with the discovery of spectosis, a caspase-8–dependent death program in mature red blood cells (RBCs) [6] (Fig. 1B). Despite lacking nuclei, mitochondria, and conventional signaling architecture, RBCs were shown by Chen et al. to undergo regulated death during complement-mediated hemolysis. Since keratinocytes lack nuclei and mitochondria, before entering the death process of keratinization or formation of the cornified envelope [7], it would be of interest to investigate if keratinocytes undergo spectosis too. Following membrane attack complex (MAC) formation, RBCs activate a truncated inflammasome component, miniNLRP3, which assembles with ASC (apoptosis-associated speck-like protein containing a caspase recruitment domain) to drive caspase-8 activation. Caspase-8, in turn, cleaves β-spectrin, dismantling the cytoskeletal lattice that maintains the erythrocyte’s biconcave shape. This biochemical cascade produces a characteristic morphological sequence—from discocytes to echinocytes, spherocytes, and finally ghosts. Pharmacologic inhibition of caspase-8 halts this progression and significantly reduces hemolysis, showing that spectrin proteolysis is the central execution event. Spectosis thus reveals an organelle-independent form of programmed death and expands the repertoire of RCD into the realm of enucleated cells, with important implications for autoimmune hemolytic anemia and paroxysmal nocturnal hemoglobinuria.

A third modality, floatptosis, arises not from sterile stress but from bacterial manipulation of host trafficking systems [8] (Fig. 1C). A virulent *Bergeyella cardium* variant (BCV) induces dramatic cytoplasmic vacuolization in macrophages, generating large single-membrane vacuoles derived from late endosomes and lysosomes. This process depends on solute carrier family 9 member A9 (SLC9A9), a solute transporter that promotes vacuole fusion and luminal acidification. Vacuolization precedes membrane permeabilization and is fully recapitulated by bacterial outer-membrane vesicles or transfected β-barrel proteins, indicating that floatptosis is driven by pathogen-derived signals rather than intrinsic stress. The sodium transport inhibitor amiloride markedly suppresses vacuole formation and enhances host resistance. Unlike paraptosis, triaptosis, or methuosis, floatptosis is defined by pathogen-controlled lysosomal fusion rather than organelle swelling alone. It may therefore represent an evolved microbial strategy to disable phagocytes and facilitate persistence.

The final modality described in 2025, mitoxyperilysis, arises when innate immune activation intersects with metabolic deprivation [9] (Fig. 1D). Wang et al. revealed that neither toll-like receptor stimulation nor carbon starvation alone is lethal to macrophages, but together they produce sustained glutathione depletion, ROS accumulation, and prolonged physical apposition of mitochondria to the plasma membrane. These mitochondria–plasma membrane (Mito-PM) contact sites become foci of lipid peroxidation—termed mitoxyperiosis—which progresses to membrane rupture during mitoxyperilysis. This pathway operates independently of caspases, gasdermins, mixed lineage kinase domain-like protein, or ferroptotic regulators and instead requires Bcl-2–associated X protein (BAX)/Bcl-2 homologous antagonist/killer 1 (BAK1)–dependent mitochondrial dysfunction and mechanistic target of rapamycin complex 2 (mTORC2)–regulated cytoskeletal control of mitochondrial positioning. Modulating mechanistic target of rapamycin (mTOR) activity reduces the duration of Mito-PM contacts and protects against lysis, suggesting therapeutic potential in cancer settings where metabolic stress and inflammatory stimuli converge.

Viewed collectively, these four studies broaden the conceptual scope of RCD by demonstrating that sodium influx, spectrin cleavage, lysosomal fusion, and mitochondria–membrane engagement can each serve as distinct executional logics. Rather than variants of a shared framework, they reveal that cells possess multiple, orthogonal strategies for terminating viability, shaped by the particular ecological pressures they face—osmotic imbalance, complement attack, microbial toxins, or inflammatory metabolic stress. This diversity likely reflects both evolutionary specialization and the structural complexity of intracellular architecture, where ion channels, cytoskeletal networks, endomembrane systems, and mitochondrial topology provide different vulnerable points that can be co-opted into death pathways.

The discoveries of 2025 challenge the notion that RCD consists of a finite and clearly delineated set of pathways [4]. These newly defined modalities demonstrate that cells can repurpose components of classical death regulators yet channel them into fundamentally different execution routes. Rather than fixed entities, cell death programs appear to form an evolving landscape of stress-integration mechanisms [10]. Determining how these modalities intersect, whether hybrid forms exist, and how their molecular signatures might be selectively targeted will be major questions for the years ahead. Together, NECSO, spectosis, floatptosis, and mitoxyperilysis highlight the remarkable adaptability of the dying cell and underscore the continually expanding frontier of RCD biology.
